# Mutations in the isocitrate dehydrogenase 2 gene and *IDH1* SNP 105C > T have a prognostic value in acute myeloid leukemia

**DOI:** 10.1186/2050-7771-2-18

**Published:** 2014-10-08

**Authors:** Kerstin Willander, Ingrid Jakobsen Falk, Roza Chaireti, Esbjörn Paul, Monica Hermansson, Henrik Gréen, Kourosh Lotfi, Peter Söderkvist

**Affiliations:** 1 Department of Clinical and Experimental Medicine, Linköping University, Linköping, Sweden; 2 Department of Medical and Health Sciences, Linköping University, Linköping, Sweden; 3 Department of Hematology, County Council of Östergötland, Linköping, Sweden; 4 Division of Hematology, Department of Medicine, Karolinska Institutet, Huddinge, Stockholm, Sweden; 5 Department of Immunology, Genetics and Pathology, Rudbeck Laboratory, Uppsala University, Uppsala, Sweden; 6 Department of Forensic Genetics and Forensic Toxicology, National Board of Forensic Medicine, Linköping, Sweden; 7 Department of Clinical Genetics, County Council of Östergötland, Linköping, Sweden

**Keywords:** AML, *IDH1*, *IDH2*, SNP, Prognostic markers

## Abstract

**Background:**

The isocitrate dehydrogenase (*IDH1/IDH2*) genes are metabolic enzymes, which are frequently mutated in acute myeloid leukemia (AML). The enzymes acquire neomorphic enzymatic activity when they mutated.

**Methods:**

We have investigated the frequency and outcome of the acquired *IDH1/IDH2* mutations and the *IDH1* SNP 105C > T (*rs11554137*) in 189 unselected *de novo* AML patients by polymerase chain reaction amplification followed by direct sequencing. The survival are presented in Kaplan Meier curves with log rank test. Multivariable survival analysis was conducted using Cox regression method, taking age, risk group, treatment, *IDH1/2* mutations and *IDH1* SNP105 genotype into account.

**Results:**

Overall, *IDH1/2* mutations were found in 41/187 (21.7%) of the AML patients. *IDH1* codon 132 mutations were present in 7.9%, whereas *IDH2* mutations were more frequent and mutations were identified in codon 140 and 172 in a frequency of 11.1% and 2.6%, respectively. The SNP 105C > T was present in 10.5% of the patients, similar to the normal population. A significantly reduced overall survival (OS) for patients carrying *IDH2* codon 140 mutation compared with patients carrying wild-type *IDH2* gene (p < 0.001) was observed in the intermediate risk patient group. Neither in the entire patient group nor subdivided in different risk groups, *IDH1* mutations had any significance on OS compared to the wild-type *IDH1* patients. A significant difference in OS between the heterozygous SNP variant and the homozygous wild-type was observed in the intermediate risk *FLT3* negative AML patients (p = 0.004).

**Conclusions:**

Our results indicate that AML-patients with *IDH2* mutations or the *IDH1* SNP 105C > T variant can represent a new subgroup for risk stratification and may indicate new treatment options.

## Background

Acute myeloid leukemia (AML) is a hematological malignancy caused by acquired genetic alterations in genes affecting the normal proliferation and terminal differentiation of myeloid progenitor cells. Based on cytogenetic abnormalities, cases of AML are usually classified into three groups, with favorable, intermediate and adverse prognosis [1]. The largest group is the intermediate risk group in which patients with cytogenetically normal karyotype (CN-AML) constitute about 45% of *de novo* AML [2,3]. These patients form a heterogeneous group where some achieve complete remission and become long term survivors, while others rapidly relapse, often with a more aggressive or resistant disease. The overall 5-year survival is 35-40%, but less than 15% in AML patients above the age of 60 [4]. During the last decades, several new mutations with prognostic impact have been identified in AML. These include internal tandem duplications (ITDs) in the fms-like tyrosine kinase 3 (FLT3) gene, conferring an adverse prognosis, and nucleophosmine 1 (NPM1) gene mutations, which in the absence of *FLT3*-ITD confer a favorable prognosis [5-8]. Both these genes have become clinically established prognostic markers in CN-AML. However, there is still a large group of intermediate risk patients without *FLT3*-ITD/*NPM1* mutations or other reliable prognostic markers, highlighting the need for additional markers that could explain the differential outcome in this heterogeneous patient group.

Genome-wide analysis in patients with AML have identified further genetic markers, thus extending the possibilities for more accurate prognostic distinctions between subgroups, and might aid the clinicians in treatment decisions such as choice of chemotherapy regime or early stem cell transplantation (SCT).

The isocitrate dehydrogenase (*IDH*) 1 and 2 genes were identified to be mutated in AML [9]. The IDH family consists of three isoforms, IDH1, IDH2 and IDH3 where IDH1 is located in the cytosol, while IDH2 and IDH3 are located in the mitochondrion and are normally involved in citrate metabolism in the tricarboxylic acid cycle [10]. The IDH1 and IDH2 enzymes are encoded by the *IDH1* gene at chromosome 2q33 and the *IDH2* gene resides at chromosome 15q26. The enzymes are NADP^+^-dependent to catalyze isocitrate oxidation to α-ketoglutarate (α-KG) and the cofactor NADPH is generated. Mutations in the *IDH1* genes were first identified in malignant gliomas [11,12] and subsequently *IDH1* mutations were frequently found in AML [9] and later also recurrent *IDH2* mutations were found in AML [13-15]. No mutations have been reported in the *IDH3* gene. *IDH1*/2 mutations are usually heterozygous with one wild-type allele and one mutant allele, affecting the arginine at codon 132 in exon 4 in the *IDH1* gene, codon 140 and codon 172 in exon 4 in the *IDH2* gene. The mutants acquire neomorphic enzymatic activity by converting α-KG to 2-hydroxyglutarate (2-HG) [16,17]. Studies have shown that *IDH1/2* mutations are associated with epigenetic alterations, by inhibiting the function of TET2, a DNA demethylase enzyme which activity is dependent on α-KG and essential for DNA demethylation. Mutations in the *IDH1* or *IDH2* genes favour 2-HG production and decrease the amount of α-KG, resulting in a hypermethylation phenotype and impaired hematopoietic differentiation [18,19]. Further, a synonymous single nucleotide polymorphism (SNP) (*rs11554137*) located in codon 105 in exon 4 in the *IDH1* gene, was recently reported to be of prognostic value in both adult and paediatric AML patients [20,21].

In this study we aimed to investigate the frequency of the acquired *IDH1* and *IDH2* mutations and the SNP 105C > T (*rs11554137*) located in the *IDH1* gene and correlate the different genotypes to the outcome in AML patients.

## Results

### *IDH1* and *IDH2* mutation analysis

All patients were successfully genotyped for *IDH1* codon 132 mutations, *IDH2* codon 140 and codon 172 mutations, and for the *IDH1* codon 105 SNP (*rs11554137*) (Table 1). Mutational data distributions in the entire cohort and in patient subgroups are presented in Table 2. In total, *IDH1/2* mutations were found in 41/189 (21.7%) of the AML patients. Fifteen patients (7.9%) had mutations in codon 132 in the *IDH1* gene resulting in four different amino acid exchanges, arg > his (7/15), arg > cys (6/15), arg > leu (1/15) and arg > gly (1/15). *IDH2* mutations were found in exon four at codon 140 in 21 (11.1%) of the patients and at codon 172 in 5 (2.6%) of the patients. For *IDH2* codon 140 mutations, two amino acid exchanges were detected: arg > gln (20/21) and arg > gly (1/21). For *IDH2* codon 172 mutations all were arg > lys exchanges (5/5). Mutations in the *IDH1* gene were mutually exclusive with mutations in the *IDH2* gene (Table 1).

**Table 1:** ***IDH1 *and *IDH2 *mutations and SNP 105C** > **T in 189 AML patients**

**Gene**	**Nucleotide change**	**Amino acid change**	**Number of patients**
*IDH1*	CGT > TGT	R132C	7
*IDH1*	CGT > CAT	R132H	6
*IDH1*	CGT > GGT	R132G	1
*IDH1*	CGT > CTT	R132L	1
*IDH2*	CGG > CAG	R140Q	20
*IDH2*	CGG > GGG	R140G	1
*IDH2*	AGG > AAG	R172K	5
*IDH1*	GGC > GGT	G105G	20

**Table 2:** **Patient characteristics and distributions of *IDH *mutations for all patients and within groups**

**CHARACTERISTIC**	**All**	***IDH1 *codon 132:**		***IDH1 *codon 105 (synonymous SNP):**		***IDH2 *codon 140:**		***IDH2 *codon 172:**	
**Total n = 189**		***Wild type***	***Mutation***	***Wild type***	***Variant***	***Wild type***	***Mutation***	***Wild type***	***Mutation***
Age median (range), years	64 (19–88)	63 (19–85)	70 (30–88)*	64 (19–88)	66 (29–84)	63 (19–88)	66 (37–83)*	64 (19–88)	72 (46–74)*
Gender									
Male	95	90	5	85	10	85	10	92	3
Female	94	84	10	84	10	83	11	92	2
Karyotype									
Normal	108	99	9	95	13	96	12	106	2
Aberrant	75	69	6	68	7	66	9	72	3
Missing data	6	6		6		6		6	
Risk group									
Low	32	31	1	30	2	29	3	31	1
Intermediate	87	80	7	72	15	75	12	86	1
High	55	51	4	52	3	51	4	52	3
Missing data	15	12	3	15		13	2	14	1
FLT3 status									
FLT3 wild type	116	109	7	104	12	101	15	114	2
FLT3-ITD	37	34	3	32	5	33	4	36	1
Missing data**	36	31	5	33	3	34	2	34	2
NPM1 status									
NPM1 wild type	99	92	7	87	12	88	11	96	3
NPM1 mutation	52	49	3	47	5	44	8	52	0
Missing data**	38	33	5	35	3	36	2	36	2
Induction treatment response									
Complete remission	132	122	10	119	13	119	13	127	5
Not complete remission	49	44	5	43	6	43	6	49	0
Missing information	8	8	0	7	1	6	2	8	0
*IDH1* codon 132									
Wild type	174			157	17	153	21	169	5
Mutation	15			12	3	15	0	15	0
*IDH1* codon 105 (synonymous SNP)									
Wild type	169					151	18	165	5
Variant	20					17	3	19	1
*IDH2* codon 140									
Wild type	168							163	5
Mutation	21							21	0
*IDH2* codon 172									
Wild type	184								
Mutation	5								

*Mann–Whitney test for difference in age distribution between patients with *IDH* mutation (*IDH1* or *IDH2*) and *IDH* wildtype patients, median 62 vs 69 years; p = 0.036.

**FLT3-ITD and *NPM1* mutations were not routinely analyzed in all non-normal karyotype patients.

No significant differences between *IDH* genotype groups in terms of median age at diagnosis, gender, treatment regime, or distribution of *FLT3/NPM1* mutations were found in the patient cohort. However, the median age at diagnosis appear to be higher in patients with mutated *IDH* gene (*IDH1* or *IDH2*) than in patients with wild-type *IDH* gene (69 vs. 62 years, respectively, p = 0.036, Table 2).

### Impact of *IDH1* and *IDH2* mutations on treatment response and overall survival

We found no significant difference on OS for patients with *IDH1* codon 132 mutations, neither in the entire group nor when stratified in different risk groups.

**Figure 1:**
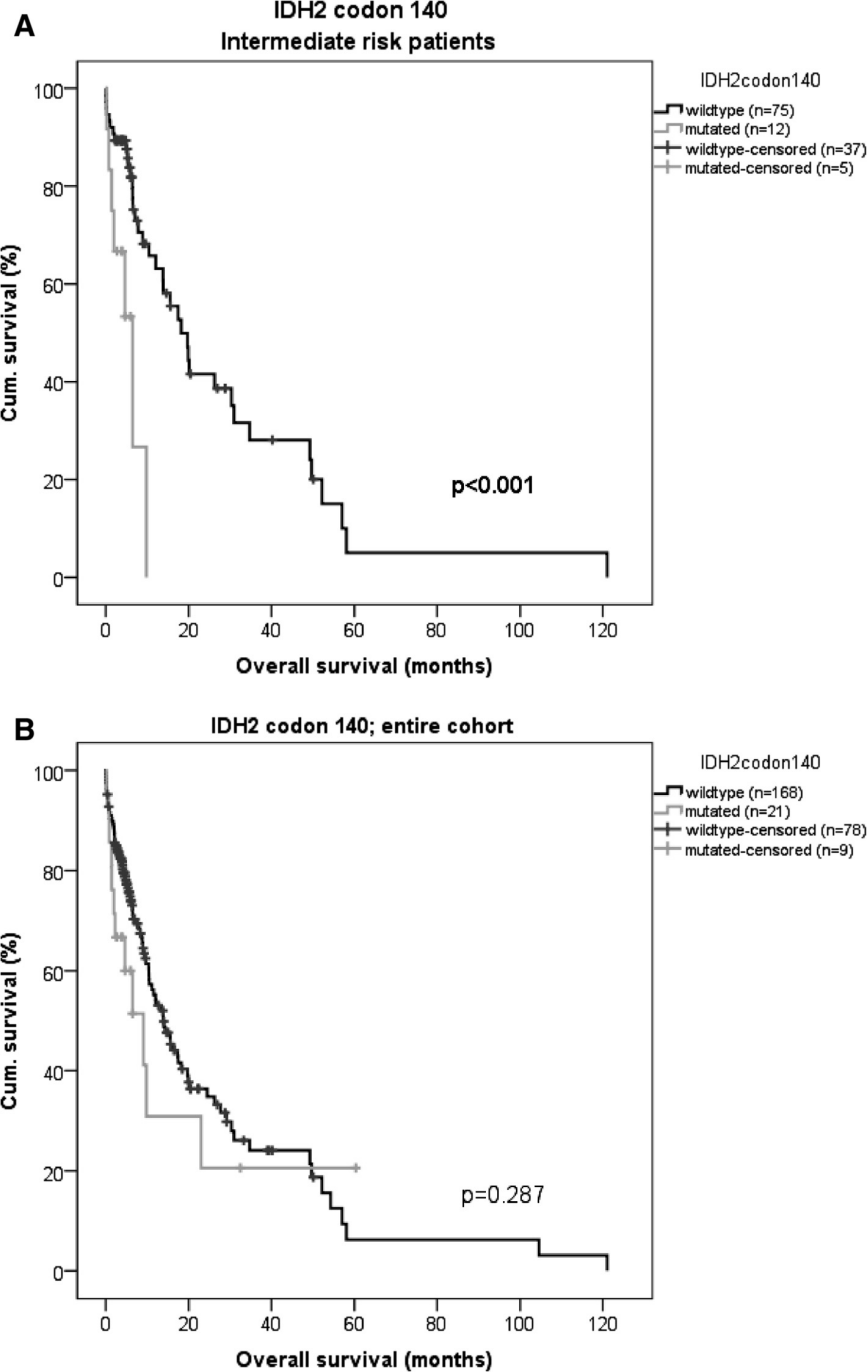
**Kaplan-Meier curves of OS, *IDH2 *codon 140. (A)** Significant differences in OS between *IDH2* codon 140 genotypes in intermediate risk AML patients. Median OS 6 *vs.* 18 months (p < 0.001) for *IDH2* codon 140 mutated patients and wild-type patients, respectively. **(B)** OS for AML-patients with mutated or unmutated *IDH2* codon 140 in the entire group, median OS 9 *vs.* 14 months, respectively (p = 0.278).

**Figure 2:**
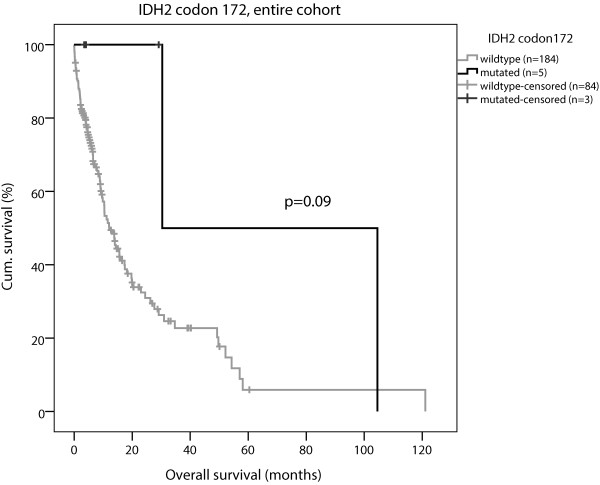
**Kaplan-Meier curve of OS, *IDH2 *codon 172.** The low frequency of AML-patients with mutated *IDH2* codon 172 showed a tendency towards improved OS survival compared to wildtype *IDH2*, OS 30 *vs.* 12 months, p = 0.09.

Mutations in the *IDH2* gene codon 140 revealed a significant increased risk for shorter OS in the whole patient group in relation to the wild type *IDH2* codon 140, (HR = 1.94; 1.07-3.53; 95% confidence interval, p = 0.03) (Cox regression Table 3; 15 patients with missing karyotype data where excluded from the analysis). This was most pronounced among the intermediate risk group patients with a median OS 6 *vs.* 18 months, for mutated and wild type patients, respectively, p = 0.001, (Figure 1A; entire cohort presented in Figure 1B). Patients with *IDH2* codon 172 mutations showed an improved survival in the entire patient group compared to patients with wild type *IDH2* codon 172 in cox regression analysis (HR = 0.22; 0.07-0.74; 95% confidence interval, p = 0.014) (Table 3) and Kaplan Meier analysis, (p = 0.09, Figure 2).

**Table 3:** Cox regression of overall survival, forced entry method

***Covariates***	***N***	***HR***	***95% CI***	***p***
Age		1.022	1.002-1.042	**0.033**
Risk group				
Low risk	32	1		
Intermediate risk	87	2.980	1.495-5.942	**0.002**
High risk	55	5.993	2.912-12.333	**<0.001**
Treatment				
Chemotherapy	118	1		
Chemotherapy + allo-SCT	56	0.231	0.118-0.450	**<0.001**
*IDH1* codon 132				
Wild type	162	1		
Mutated	12	0.816	0.390-1.708	0.59
*IDH2* codon 140				
Wild type	155	1		
Mutated	19	1.942	1.068-3.530	**0.030**
*IDH2* codon 172				
Wild type	169	1		
Mutated	5	0.222	0.067-0.738	**0.014**
*IDH1* SNP codon 105 GGC > GGT				
Wild type	154	1		
Variant	20	1.496	0.812-2.756	0.196

95% CI = 95% Confidence interval; HR = Hazard ratio.

Significant *P*-values (*P* <0.05) in boldface.

There were no significant differences in the distribution of *IDH1* or *IDH2* genotypes among patients with CR and no CR.

### The *IDH1* SNP variant influences overall survival

**Figure 3:**
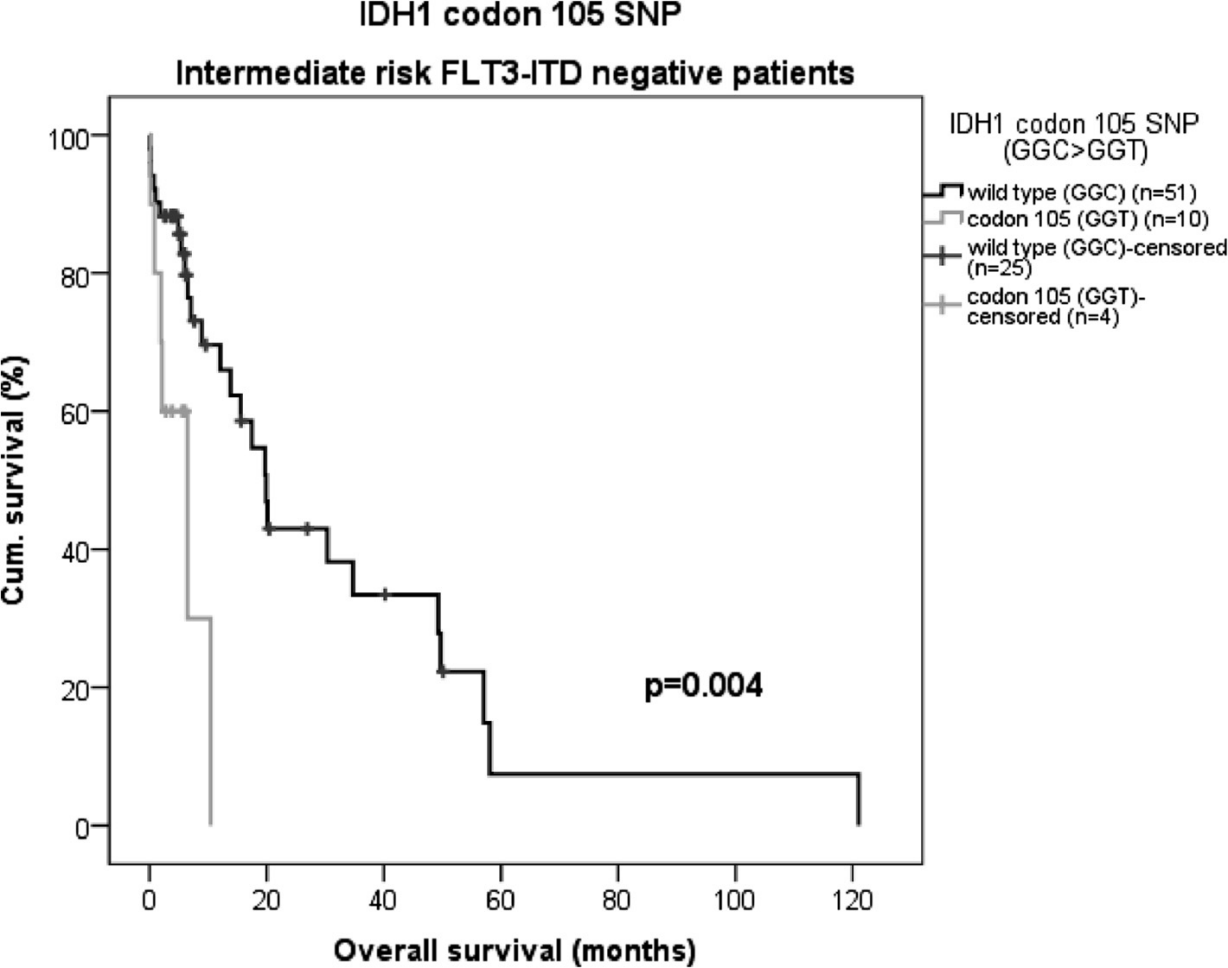
**Kaplan-Meier curve of OS, *IDH1 *codon 105 SNP.** Significant differences in OS between codon 105 genotypes in *FLT3*-ITD negative intermediate risk patients; median OS 6 *vs*. 20 months (p = 0.004) for codon 105 variant allele and wild-type patients, respectively.

All patients were successfully genotyped for *IDH1* codon 105 SNP (*rs11554137*) (Table 1) that was not associated with the *IDH* mutations (only 7 overlapping cases; 3 in *IDH1* and 4 in *IDH2*). The synonymous SNP (GGC > GGT) was found in 20 patients (10.6%) in the entire cohort. Kaplan Meier curves with log rank tests also revealed a significant difference in OS between the *IDH1* codon 105 SNP variants, where heterozygous carriers of the T allele displayed a shorter survival compared to patients with homozygous wild-type C alleles. This was significant only in the intermediate risk *FLT3*-ITD negative AML patients. In this risk group, the median OS was 20 *vs.* 6 months for codon 105 wild-type C/C and variant T/C patients, respectively, (p = 0.004, Figure 3). It should be noted that all the intermediate risk *FLT3*-ITD negative patients with the codon 105 T allele were also negative for *NPM1* mutations. However, in cox regression analysis the codon 105 SNP did not display independent significance due to other stronger factors affecting the outcome in the entire cohort (Table 3).

## Discussion

Mutations in the *IDH1* and *IDH2* genes in AML are reported as being associated to diverse outcomes by different groups [22]. Mardis *et al*. [9] was the first to identify mutations in the *IDH1* gene as a new recurrent mutation associated with CN-AML. Further, Marcucci *et al*. [13] reported two different mutations in the *IDH2* gene (R140 and R172) in AML. In the present study we have investigated the frequency and impact of *IDH* mutations on outcome according to the different clinical risk groups, normal/aberrant cytogenetics, and also according to the *FLT3* and *NMP1* mutation status in 189 unselected AML patients. In our study cohort we found *IDH1* and *IDH2* mutated in 21.7% of the cases. *IDH2* mutations were more common than *IDH1* mutations (13.8% vs 7.9%). The frequency of *IDH1* mutations (7.9%) in our AML cohort is similar to previous reports of unselected AML patients (5.5%-10.4%) [9,14,15,17,23-26]. The *IDH2* mutations have been reported to have a prevalence of 6.1%-17.7% in unselected AML [15,17,24-28], as compared to 13.8% in our study group. Investigation of the influence on OS in the entire study population (no selection in karyotypes, risk groups or *FLT3/NPM1* status) for *IDH1* mutations conferred no significant difference compared to wild-type *IDH1*, nor when statistical stratification was applied. In some studies, an influence on OS is seen with *IDH1* mutations for patients with CN-AML or intermediate risk group according to the *FLT3/NPM1* status [13-15,23,28], while other groups could not detect any impact on survival with mutated *IDH1* gene, which is in accordance with our results [9,20,24,26]. Furthermore, in our cohort we also found a slightly higher median age at diagnosis in patients with mutated *IDH1* gene than in patients with wild type *IDH1*. Our cohort covered a wide age span including both younger and older patients, but future studies investigating the impact of *IDH* mutations specifically in older AML patients could be warranted.

Two hotspot mutations are found in exon 4 in the *IDH2* gene, R140 and R172. In our cohort we found R140 to be altered with a frequency of 21/189 (11.1%) and R172 in 5/189 (2.6%) patients. We found prognostic significance on OS for the *IDH2* codon 140 mutations, where the intermediate risk patients with codon 140 mutations revealed a significantly shorter OS than codon 140 wildtype. *IDH2* codon 172 mutations were identified in a low frequency, only in 5 individuals, and were provided with a favorable outcome in our study cohort. However, Ward *et al.* noted a trend toward improved survival for patients with *IDH2* codon 140 mutations and also Green *et al.* reported an unexpected favorable outcome associated with *IDH2* R140 mutations and an unfavorable outcome for patients with *IDH2* R172 mutation [17,27]. Patel *et al.* also found a favorable effect for patients with mutant *IDH2* codon 140 [29]. However, in the study of Green *et al.*, there was a difference in patient median age at diagnosis compared to our study cohort, 43 vs. 64 years respectively. In the study of Patel *et al.* the patients’ median age at diagnosis also was much lower than in our cohort, 48 vs. 64 years respectively. These may indicate that the effect of the *IDH2* mutations is seen in elderly patients.

Figueroa *et al.*[18] have shown that IDH mutant enzymes may result in a global DNA hypermethylation profile, blocking the cellular differentiation in hematopoietic cells through inhibition of demethylation of 5-metylcytosine (5-MeC) mediated via TET2. TET2 require α-KG for demethylation of 5-MeC and, other studies have demonstrated that the mutation dependent metabolite 2-HG is sufficient to promote leukemogenesis when mutated *IDH1* and *IDH2* convert α-KG to 2-HG [30]. The *TET2* gene is also identified to be mutated in de novo AML (7-23%) and is mutually exclusive with *IDH1/2* mutations [31].

The synonymous SNP 105C > T, located in the same exon, but only overlapping in three cases, as R132 in the *IDH1* gene, was also analyzed in our study. *IDH2* mutations simultaneously with the codon 105 variant were found in three patients with codon 140 mutation and in one case with codon 172 mutation. The frequency of the SNP was 20/189 (10.6%) in the entire cohort, and almost the same frequency (11.7%) has been reported among healthy volunteers by a German group [20]. The same group also reported this SNP to correlate to an inferior prognosis in CN-AML [20]. In accordance with this, we found a pronounced significant inferior overall survival in intermediate risk *FLT3*-ITD negative patients carrying the variant codon 105 allele. The biologic effect of the silent SNP remains to be investigated in AML. One speculative explanation with a synonymous SNP is that it will cause a change in the rate of the protein translation resulting in affected protein folding and altered function of the protein [32], or cause a new splicing site. Potentially the T variant enables a new splice site (GTGG[C/T]ACGG > GTGgtacgg), resulting in a possible mRNA difference of 100 bp. Calculation of splice-site scores by using the Analyzer Splice Tool (http://ibis.tau.ac.il/ssat/SpliceSiteFrame.htm) would give a score of 72.5 to the potentially new splice site with the T variant compared with the natural splice site at the end of exon 4, which gives a score of 88.1. To test this possibly new splice site, we sequenced cDNA in three patients from this study with the SNP T allele, but the results provided no difference in sequence length between the C or T alleles, and thus no new splice variant was detected.

In summary, our results identified in total 21.7% *IDH1/IDH2* mutations in the study population. Our results indicate that the *IDH2* codon 140 mutation have the highest potential as a prognostic marker, further stratifying intermediate risk patients.

In addition, the synonymous SNP 105C > T in the *IDH1* gene may be a novel prognostic marker in AML of intermediate risk *FLT3* negative patients however, this has to be confirmed through future studies. These markers may be especially useful in this heterogeneous group of AML patients, where other prognostic markers are absent and the outcomes vary widely. Further, studies have been carried out on possible new drugs by targeting the mutant IDH enzyme on leukemia cells, resulting in inhibition of accumulation of the 2-HG oncometabolite and subsequently differentiation of the AML blasts [33,34].

## Conclusions

*IDH* mutational status and/or *IDH1* SNP 105C > T variant may represent a new subgroup of AML patients and have the potential as tools for selecting patients expected to benefit the most from the new treatment alternatives.

## Methods

### Patients

This study included 189 Swedish patients diagnosed with *de novo* AML at Linköping University Hospital and Karolinska University Hospital in Huddinge between 1988 and 2010. The inclusion of the patients in this study was not consecutively included. Median age at diagnosis was 64 years, range 19–88 years. Patient characteristics are summarized in Table 2. Bone marrow or peripheral blood samples collected at diagnosis were used to isolate DNA for further genetic analysis. Risk group assignment at diagnosis was based on cytogenetic and molecular genetic findings as defined by ELN (European Leukemia Net) [35] and International Working Group recommendations [36], and other prognostic factors such as age, performance status and comorbidity, with minor modifications (see Swedish Hematology Association guidelines, http://www.sfhem.se/Filarkiv/Nationella-riktlinjer accessed 2013-05-28). Swedish AML patients diagnosed in 2004 or later have been treated according to nationwide AML treatment guidelines (http://www.sfhem.se/Filarkiv/Nationella-riktlinjer, accessed 2013-05-28). Thus, the majority of the patients received induction treatments including daunorubicin 60 mg/m^2^ once a day for three days combined with Cytarabine (AraC) as 1000 mg/m^2^ twice a day in 2 h i.v. infusions for 5 days. Before 2004, regional guidelines most commonly included AraC doses of 200 mg/m^2^ as 24 h i.v. infusions for 7 days combined with three days either daunorubicin or idarubicin [37]. Some patients also received other drugs in combination with daunorubicin/idarubicin and/or AraC, such as Mitoxantrone, Etoposide, 6-Thioguanine and Cladribine. For further treatment details, see Table 4. Treatment response was evaluated as non-complete remission (no CR) or morphologic complete remission (CR) [36]. Patients treated by allogeneic stem cell transplantation (allo-SCT) (n = 59) were censored at the time of transplantation in the survival analysis. Informed consent was obtained from the patients and the study was approved by the local ethical committees and conducted in accordance with the Helsinki declaration.

**Table 4:** Induction treatment regimes

**Regime**	**N (%)**
Daunorubicine and Cytarabine (n = 116) or Daunorubicine, cytarabine and mitoxantrone (n = 2)	118 (62.4)
Idarubicine and Cytarabine (n = 26) or Idarubicine, Cytarabine and Etoposide (n = 3)	29 (15.3)
Idarubicine, Cytarabine and Cladribine	20 (10.6)
Mitoxantrone, cytarabine and Etoposide (n = 7) or Mitoxantrone and Cytarabine (n = 2)	9 (4.8)
Daunorubicine, Cytarabine and 6-Thioguanine	8 (4.2)
Other/unknown^1^	5 (2.6)

^1^Including 2 in clinical trial of combination therapy with Tipifarnib, and 1 with Fludarabine, Cytarabine and G-CSF. 2 patients with unknown treatment but known curative intent.

### *IDH1* and *IDH2* genotyping analysis

Mononuclear cells from either peripheral blood or bone marrow were enriched by Ficoll-Paque density centrifugation at the time of diagnosis and genomic DNA was extracted. For *IDH1* and *IDH2* genotyping analysis, a PCR reaction was performed in a total volume of 20 μl containing 10–50 ng DNA, 0.5U Taq DNA polymerase, 2 mM MgCl_2,_ 0.2 mM dNTPs, 1xPCR buffer, 1 μM each of *IDH1* forward primer (5’ctcagagccttcgctttctg) and reverse primer (5’cacatacaagttggaaatttctgg) and of *IDH2* forward primer (5’ggggttcaaattctggttga) and reverse primer (5’ctaggcgaggagctccagt). The terminal cycling conditions for both *IDH1* and *IDH2* were an initial denaturation at 94°C for 2 min followed by 35 cycles at 94°C for 30 s, 55°C for 30 s, 72°C for 30 s and an end extension at 72°C for 5 min. The PCR product was purified by using ExoSAP-IT and direct sequencing was performed by using BigDye Terminator v3.1 Cycle Sequencing Kit (AB Applied Biosystems). The *IDH1/2* sequences were compared to the wild type *IDH1/2* to detect the genetic variations (NM_005896.2 and NM_002168.2 respectively).

### Statistical analysis

Fisher’s exact test was used to compare differences in genotype distribution between patients with CR and no CR. Mann Whitney Test or Fisher’s exact test was used to investigate differences between genotype groups in terms of age, gender and karyotype distributions, or other characteristics. Kaplan Meier survival analysis with log rank test for significance was used to evaluate the impact of *IDH1* and *IDH2* genotype on overall survival (OS) (calculated as time from diagnosis until death, date of the latest follow-up, or date of allo-SCT). Multivariable survival analysis was conducted using Cox regression with a forced entry method, taking age, risk group, treatment, *IDH1/2* mutations and *IDH1* SNP105 genotype into account. The impact of *IDH* genotype was evaluated in the entire patient material and in subgroups stratified by risk group and *FLT3* status. A p-value of 0.05 was considered significant, and all analyses were performed using IBM SPSS Statistics v.20.

## Competing interests

The authors declare that they have no competing interests.

## Authors’ contributions

K.W: Research, laboratory work, data compilation, manuscript writing; IJF: Resarch, clinical data compilation and statistical analysis, manuscript writing; R.C: Clinical data compilation and consultation; E.P: Patient material and clinical data collection; M.H: *FLT3*/*NPM1* analysis, data collection; H.G: Data and statistical analysis; K.L: Research, patient material and clinical data collection; P.S: Conception and study design, research. All authors critically reviewed the manuscript. All authors read and approved the final manuscript.

## Authors’ information

Kourosh Lotfi and Peter Söderkvist share last authorship.
